# Retraction Note: Joint detection of ERCC1, TUBB3, and TYMS guidance selection of docetaxel, 5-fluorouracil and cisplatin (DDP) individual chemotherapy in advanced gastric cancer patients

**DOI:** 10.1186/s40001-015-0134-4

**Published:** 2015-03-26

**Authors:** Yushuang Luo, Zhanquan Li, Sen Cui, Cunfang Shen, Junhui Zhao, Milu Wu, Yuying Li, Miaozhou Wang, Rong Chen, Zhibo Liu, Ge Ri-li

**Affiliations:** Affiliated Hospital of Qinghai University, Kunlong Road 16, Tongren Road 29, 810001 Xining, China; Research Center for High Altitude Medicine, Qinghai University Medical School of Medicne, 810001 Qinghai, China

## Retraction

The Publisher and Editor regretfully retract this article [1] because the peer-review process was inappropriately influenced and compromised. As a result, the scientific integrity of the article cannot be guaranteed. A systematic and detailed investigation suggests that a third party was involved in supplying fabricated details of potential peer reviewers for a large number of manuscripts submitted to different journals. In accordance with recommendations from COPE we have retracted all affected published articles, including this one. It was not possible to determine beyond doubt that the authors of this particular article were aware of any third party attempts to manipulate peer review of their manuscript.
